# Phytochemical Analysis, Antioxidant Potential and Antibacterial Activities of Different Anatomical Parts of *Hypericum cordifolium* Choisy

**DOI:** 10.1155/2024/8128813

**Published:** 2024-05-25

**Authors:** Sujana Sapkota, Alishma Maharjan, Sanjeeta Tiwari, Meena Rajbhandari

**Affiliations:** ^1^Department of Chemistry, Tri-Chandra Campus, Tribhuvan University, Kirtipur, Nepal; ^2^Natural Products Laboratory, Research Centre for Applied Science and Technology, Tribhuvan University, Kathmandu 44600, Nepal

## Abstract

The genus *Hypericum* comprises a large number of species. The flower, leaf, stem, and root of the *Hypericum* species are widely used in traditional medicine in different cultures. Many *Hypericum* species have been well investigated phytochemically and pharmacologically. However, only a few reports are available on the *H. cordifolium* native to Nepal. The present study aims to evaluate the phytochemical composition of different extracts, qualitative analysis of methanol extract of the flower and leaf using thin-layer chromatography (TLC), and the antioxidant properties of components by the TLC-DPPH^.^ assay. The phenolic and flavonoid contents were estimated in different extracts of the leaf and stem, and their antioxidant and antibacterial activities were evaluated. In the phytochemical screening, phenolics and flavonoids were present in ethyl acetate, methanol, and 50% aq methanol extracts of both the leaf and stem. In TLC analysis, the methanol extract of flowers showed the presence of 11 compounds and the leaf extract showed the presence of 8 compounds. Both extracts contained chlorogenic acid and mangiferin. Hyperoside and quercetin were present only in the flower extract. In the TLC-DPPH^.^ assay, almost all of the flower extracts and 5 compounds of the leaf extract showed radical scavenging potential. Estimation of phenolics and flavonoids showed that all the leaf extracts showed higher amounts of phenolics and flavonoids than stem extracts. Among leaf extracts, greater amounts of phenolics were detected in 50% aqueous methanol extract (261.25 ± 1.66 GAE/g extract) and greater amounts of flavonoids were detected in methanol extract (232.60 ± 10.52 CE/g extract). Among stem extracts, greater amounts of flavonoids were detected in the methanol extract (155.12 ± 4.30 CE/g extract). In the DPPH radical scavenging assay, the methanol extract of the leaf showed IC_50_ 60.85 ± 2.67 *µ*g/ml and 50% aq. methanol extract of the leaf showed IC_50_ 63.09 ± 2.98 *µ*g/ml. The methanol extract of the stem showed IC_50_ 89.39 ± 3.23 *µ*g/ml, whereas ethyl acetate and 50% aq. methanol extract showed IC_50_ > 100 *µ*g/ml. In the antibacterial assay, the methanol extract of the leaf showed the inhibition zone of 12-13 mm and the stem extract showed the inhibition zone of 7–11 mm against *S. aureus*, *E. coli*, and *S. sonnei*, whereas both extracts were inactive against *S. typhi*. The findings of this study support the traditional use of this plant in Nepal for the treatment of diseases associated with bacterial infections. The present study revealed that the underutilized anatomical parts of *H. cordifolium* could be the source of various bioactive phytochemicals like other *Hypericum* species.

## 1. Introduction

The genus *Hypericum* is one of the most important medicinal plants that comprise 484 species widely distributed in tropical, temperate, and high mountains around the world [1]. Among them, *H. perforatum* is one of the well-studied species. In Germany, the flowers, leaves, stems, and roots are widely used in traditional medicine [2]. It is available in pharmacies as licensed medicine for the treatment of anxiety and mild to moderate depression. In Britain, products prepared from *H. perforatum* are consumed as traditional herbal medicines. In the USA, it is used as a dietary supplement and has very high demand [3, 4]. Because of the high economic value of this plant to the herbal industry, it is cultivated in addition to wild collection [5]. Generally, the dry extract of the aerial part of the herb is applied for internal use and the oily extract is used for external application [6].

The effectiveness of *H. perforatum* extracts to treat depression has been studied in several clinical trials. The results were positive in comparison to placebo. It has a similar effect to standard antidepressants [7, 8]. In addition, it showed anti-inflammatory, hepatoprotective, antiviral, antimicrobial, antioxidant, antitumor, and wound healing activities [9–11]. The observed activities are due to the presence of a wide range of secondary metabolites such as naphthodianthrones, acyl-phloroglucinols, flavonoids, phenolic acids, and xanthones [12, 13]. It has been reported that not only *H. perforatum* but other *Hypericum* species have also a wide range of pharmacological activities [14, 15]. Therefore, they need to be investigated phytochemically and pharmacologically.

There are many *Hypericum* species available in Nepal. In Kathmandu valley, at an altitude between 900 and 2000 m, *H. cordifolium* is one of the easily available species. Locally, it is called Areli, Areto, or Ghod Jatra Phool. The golden yellow flowers appear in March to April. The locals use the flowers for religious purposes, plant juice for menstrual disorders, root juice for diarrhea, and bark juice for back pain and dislocation of bone. Similarly, flower paste is eaten to cure dysentery, fresh young shoots to relieve throat pain, and root and flower juice to treat fever, pneumonia, diarrhea, dysentery, cough, and cold [16]. In *H. perforatum*, the bioactive secondary metabolites accumulate in the aerial parts, so herbal products are usually prepared from leaves and flowers [2]. This has led us to work on flower, leaf, and stem extracts of *H. cordifolium* separately. In our previous work, we have investigated flower extracts for wound healing activities, some phytochemicals such as polyprenylated aromatic acylphloroglucinols were isolated, and phytochemical analysis, antioxidant, antibacterial, and cytotoxic activities were evaluated [17–19]. The aim of the present work was to evaluate the phenolic composition of leaf and flower extracts by thin-layer chromatography, TLC, and antioxidant properties of components of the extracts by the TLC-DPPH^.^ assay. Furthermore, the contents of phenolics and flavonoids in different leaf and stem extracts were quantified, and their antioxidant and antibacterial activities were determined. The findings of this study support the traditional medical knowledge of the local people to some extent and utilization of the underexplored natural resources of Nepal for the formulation of herbal drugs or nutraceuticals for primary care.

## 2. Materials and Methods

### 2.1. Chemicals

All the solvents and chemicals used were of analytical grade. Gallic acid and precoated silica gel 60 GF_254_ were purchased from Merck, Darmstadt, Germany. (±)-Catechin and 2,2-diphenyl-1-picrylhydrazyl (DPPH) were purchased from Sigma Chemical Company, USA. The chemicals like Folin–Ciocalteu phenol reagent (FCR), AlCl_3_, Na_2_CO_3_, NaNO_2_, NaOH, and FeCl_3_ and solvents like n-hexane, dichloromethane, ethyl acetate, methanol, and acetone were purchased from Thermo Fisher Scientific India, Pvt. Ltd. Muller–Hinton agar (MHA) was from Himedia Laboratories Company Ltd., India. Neomycin was from Sigma-Aldrich Co. Ltd. Double distilled water was used throughout the experiments.

### 2.2. Plant Materials and Extraction

Plant materials were collected in April 2021 from the pine forest of Nagarkot area of Bhaktapur district. It was identified by Prof. Dr. R. P. Chaudhary, Research Centre for Applied Science and Technology, RECAST, Tribhuvan University. The voucher sample (#HC-21-MR) was deposited at RECAST. The separated flowers, leaves, and stems were shade dried separately and crushed to fine powder using a kitchen grinder. The crushed leaf and stem (50 g each) were extracted successively with hexane (250 ml), dichloromethane (250 ml), ethylacetate (200 ml), and methanol (200 ml) using a Soxhlet extractor. The remaining residue after extraction with methanol was dried and refluxed with 50% aqueous methanol (100 ml) for 2 hours and then allowed to cool and filter. The solvents were evaporated separately under reduced pressure using rotavapor (BUCHI R-200, BUCHI V-800). The concentrated extracts were kept in a freezer at −20°C for further use.

### 2.3. Chemical Screening

The presence of various classes of phytochemicals like polyphenols, flavonoids, tannins, terpenoids, alkaloids, saponins, quinones, glycosides, and reducing sugars were tested by using different specific reagents. The standard protocol of Culie was adopted for the detection of phytochemicals [20].

### 2.4. Optimization of TLC Separation and TLC-DPPH Assay

The powdered leaves and flowers of *H. cordifolium*, each 1 g, were first extracted with dichloromethane and then with methanol at room temperature. The extracts were analyzed by thin-layer chromatography (silica gel 60F_254_ Merck, 11 × 2 cm plate, run time 30 min), and different combinations of mobile phases (no. 1–5) were used [19, 21]. The chromatograms were developed with different mobile phases to optimize the chromatographic conditions. The developed plates were dried at room temperature, and the bands were visualized under a UV lamp (DESAGA, HP-UVIS) at 254 and 366 nm. Subsequently, the plate was sprayed with the chromogenic agent, FeCl_3_. For the determination of *R*_*f*_ and *λ*^max^ values of each band, preparative TLC separation was carried out on the optimized mobile phase in silica gel 60 F_254_ aluminum plate (10 × 5 cm). The major bands were cut carefully and extracted with methanol.

For TLC-DPPH^.^, the chromatogram developed in an optimized mobile phase was sprayed with 0.2% methanolic DPPH solution and allowed to dry. The antioxidant activities of different components were observed as yellow spots on a purple background [22].

1. Ethyl acetate-methanol-water (100 : 13.5 : 10, v/v/v)
2. Ethyl acetate-acetic acid-formic acid-water (100 : 11 : 11 : 26, v/v/v/v)
3. Ethyl acetate-acetic acid-formic acid-water (100 : 11 : 11 : 27, v/v/v/v)
4. Ethyl acetate-acetic acid-formic acid-water (72 : 7:7 : 14, v/v/v/v)
5. Ethyl acetate-formic acid-water (67 : 13 : 20, v/v/v)

### 2.5. Determination of Total Phenolic (TPC) and Total Flavonoid (TFC) Content

The total phenolic content in different leaf and stem extracts was estimated by using the Folin–Ciocalteu (FC) reagent as previously described [23]. The calibration curve was constructed using gallic acid. Various concentrations of gallic acid solutions were prepared (10, 25, 50, 75, and 100 *µ*g/mL). In a 20 mL test tube, 1 mL gallic acid solution of each concentration was added, and then 5 mL 10% FC reagent and 4 mL 7% sodium carbonate were added. The blue mixture was shaken well and incubated for 30 minutes at 40°C in a water bath. Then, the absorbance was measured at 760 nm against a blank using the SHIMADZU UV-1900I spectrophotometer. Similarly, various concentrations of the extracts (100, 50, 25, and 12.5 *µ*g/ml) were prepared. Following the procedure applied for gallic acid, absorbance for each concentration of the extract was recorded. TPC was calculated using the formula: *C* = *c* V/m, where *C* represents total phenolic content in mg GAE/g dry extract, *c* indicates the concentration of gallic acid obtained from the calibration curve in mg/mL, V indicates the volume of the extract in ml, and m is the mass of the extract in gram. It is expressed as mg gallic acid equivalents (GAE) per gram dry extract (mg/g).

The total flavonoid content in different extracts of the leaf and stem was estimated by using the aluminium chloride reagent [24]. The calibration curve was constructed using (±)-catechin. Various concentrations of catechin solutions (10, 25, 50, 75, and 100 *µ*g/mL) were prepared. In a 20 mL test tube, 1 mL catechin solution of each concentration, 6.4 mL of double distilled water, and 0.3 mL of 5% NaNO_2_ solution were added. After 5 minutes, 0.3 mL of 10% AlCl_3_ solution was added and waited for 1 minute. Then, 2 mL of 1 M NaOH was added with shaking. The absorbance of the pink mixture was determined at 510 nm using a SHIMADZU UV-1900I spectrophotometer. Similarly, various concentrations of the extracts (100, 50, 25 and 12.5 *µ*g/ml) were prepared. Following the procedure applied to catechin, absorbance for each concentration of the extract was recorded. The total flavonoid content of the extracts was calculated as described in the case of phenolics.

### 2.6. Determination of Antioxidant Activity

The antioxidant activity of the extracts and ascorbic acid was determined using the DPPH free radical as described by Brand-Williams with a slight modification [25]. DPPH solution (0.10 mM) was prepared in methanol. Plant extracts and ascorbic acid solutions of different concentrations (20, 30, 40, 50, 60, 70, 80, 90, and 100 *µ*g/ml) were prepared in methanol. To 0.5 ml of ascorbic acid or extract, 2.5 ml of DPPH solution was added with shaking and was kept in the dark for 30 min. Then, absorbance was measured at 517 nm using the SHIMADZU UV-1900I spectrophotometer. Control was prepared by adding 0.5 ml methanol instead of ascorbic acid or extract. The percentage of DPPH radical scavenging activity was calculated using formula (1), where *Ac* is the absorbance of control and *As* is the absorbance of solution. IC_50_ value was calculated from the plotted graph of radical scavenging percentage against the concentration of ascorbic acid or extracts.

(1) $$\begin{array}{c} \%\text{ of radical scavenging}=\frac{Ac-As}{Ac}\times 100. \end{array}$$

### 2.7. Determination of Antimicrobial Activity

The antimicrobial activities of the extracts were evaluated by the agar well diffusion method [26] against one Gram-positive bacterium *Staphylococcus aureus* (ATCC 25923) and three Gram negative bacteria, *Salmonella typhi* (ATCC 14028), *Escherichia coli* (ATCC25922) and *Shigella sonnei* (ATCC25931). The extracts were prepared at a concentration of 50 mg/ml in 50% DMSO. Then, 50 *μ*L of the prepared extract was introduced into the agar well of 6 mm diameter seeded with the respective microorganisms. Negative control experiments were performed using an equivalent volume of 50% DMSO, and positive control experiments were performed using a standard antibiotic, neomycin (1 mg/ml). The plates were kept in the refrigerator at 4°C for 4 hours, and then they were turned over to incubate overnight at 37°C in an inverted position. At the end of the incubation period, the clear inhibition zone of bacterial growth was observed around each well in the presence of different extracts/neomycin that was measured.

### 2.8. Statistical Analysis

For total phenolic, flavonoid content, and antioxidant activity determination, absorbance data were recorded as a mean of three determinations for different concentrations. The total content of phenolic, flavonoid, and IC_50_ values in the DPPH assay were calculated from the regression equation of the calibration curve, *Y* = *mx* + *c*, where *Y* is the absorbance of the extract, *m* is the slope from the calibration curve, *x* is the concentration of extract, and *c* is the intercept. The linear correlation coefficient (*R*^2^) values were also calculated. All the data were presented as a mean ± SD. The mean values were compared using one-way ANOVA. In the case where the results were statistically different (*p* < 0.05), the Tukey–Kramer multiple comparison test was performed using Microsoft Excel 2016.

## 3. Results and Discussion

### 3.1. Extractive Values in Different Solvents

The analysis of herbal drugs generally requires suitable extraction methods. Extraction with solvents of increasing polarities above room temperature, starting from n-hexane, dichloromethane, ethyl acetate, methanol, and 50% aqueous methanol, provided different amounts of extracts. The results are presented in Table 1. At elevated temperature, the viscosity and surface tension of the solvents are decreased. It helps the solvents reach the sample matrices which facilitate the extraction rate [27, 28]. Therefore, we have adopted the Soxhlet extraction method. In the case of the leaf, the extract yield was in the order, CH_2_Cl_2_ > MeOH > 50% aq. MeOH > EtOAc > n-hexane. In the case of the stem, the extract yield was in the order of 50% aq. MeOH > MeOH > n-hexane > EtOAc > CH_2_Cl_2_. This indicated that the yield depends on the chemical composition of the sample as well as the type of solvent used in the extraction process. For example, the leaf contains high amounts of less polar phytochemicals that are readily soluble in dichloromethane than hexane. Similarly, the stem contains large amounts of more polar phytochemicals that are readily soluble in 50% aq. methanol than methanol.

### 3.2. Screening for Phytochemicals

Plants are sources of bioactive compounds. They are used for curing as well as healing of various human diseases. Screening of the plant extracts for phytochemicals provides a great knowledge about the occurrence of specific classes of phytochemicals that has great application. Therefore, phytochemical screening was conducted. Phytochemical screening of leaf and stem extracts revealed the presence of many classes of phytochemicals such as phenolics, tannins, flavonoids, alkaloids, saponins, quinones, and terpenoids. In the hexane extracts of the leaf and stem, most of the tested phytochemicals were absent and only terpenoids and quinones were present. In the dichloromethane extract of the leaf, alkaloids, phenolics, and quinones were present, whereas in the stem extract, only phenolics and terpenoids were detected. The positive test for phenolics is due to the presence of less polar acylphloroglucinols which are characteristics of *Hypericum* species [14].

In the ethyl acetate extract of the leaf, phytochemicals like alkaloids, flavonoids, phenolics, tannins, and quinones were present, whereas in the stem extract, terpenoids, flavonoids, phenolics, and tannins were present. In the case of methanol extracts of the leaf and stem, most phytochemicals such as terpenoids, flavonoids, phenolics, glycosides, and tannins were present. In addition, the leaf extract also contains alkaloids, reducing sugars, and saponins. In the case of 50% aq. methanol extracts of the leaf and stem, terpenoids, flavonoids, phenolics, tannins, and glycosides were present. Additionally, the leaf extract also contains saponins. The phytochemical screening of leaf and stem extracts revealed that leaf extracts are richer sources of diverse classes of phytochemicals than stem extracts. This is the first report on the phytochemical screening of leaf and stem extracts. In a previous study, only the ethanol extract of the leaf was investigated [29]. The results of phytochemical screening of leaf and stem extracts of *H. cordifolium* are presented in Table 2.

### 3.3. TLC Separation and TLC-DPPH Assay

The patterns of phenolics of *H. cordifolium* flower, leaf, and stem extracts were examined by the TLC fingerprint method. The plant material was extracted first with dichloromethane at room temperature to remove nonpolar phytochemicals followed by methanol. As the aim of the present study is to detect the distribution of phenolic acids, flavonoids, and their glycosides as key marker compounds for chemical profiling, the methanol extract was chosen for TLC analysis as described by Scotti et al. [3]. In TLC analysis, the stem extract did not show detectable bands, so it was discarded. This method is useful for confirming the quality of the product. The United States and the European Pharmacopoeia adopted this method to control the quality of *H. perforatum* [30]. In TLC analysis, different mobile phase compositions were used, but better separation was observed in ethylacetate-methanol-water, 100 : 13.5 : 10 (v/v/v) with good resolution of bands as observed under UV lamp 366 nm. TLC analysis of the flower extract revealed seven bands at 254 nm, eleven bands at 366 nm, and nine bands with the chromogenic agent, FeCl_3_. The leaf extract revealed five bands at 254 nm, eight bands at 366 nm, and five bands with the chromogenic agent, FeCl_3_. Most of these bands were phenolic compounds, including flavonoids, their glycosides, and phenolic acids. The bands were identified based on the colour observed under the UV lamp at 366 nm, the *R*_*f*_ values, and the UV *λ*^max^ values [19, 31–33]. The observed results are presented in Table 3.

Band 1 (*R*_*f*_ 0.12) in both extracts corresponds to chlorogenic acid, and band 3 (*R*_*f*_ 0.40) corresponds to mangiferin. Band 4 (*R*_*f*_ 0.46) and band 11 (*R*_*f*_ 0.75) corresponding to hyperoside and quercetin, respectively, are present in the flower extract only. A bright orange band with *R*_*f*_ 0.64 was seen in the leaf extract and a very light pink band with the same *R*_*f*_ value was seen in the flower extract, which could be hypericin. In our previous work, we have reported the presence of chlorogenic acid, hyperoside, and quercetin in the flower extract [19], and hypericin and rutin were not detected in TLC [19]. The results of the TLC fingerprint analysis indicated that the phytochemical compositions of the flower and leaf extracts were different. This may lead to different biological activities of the two anatomical parts. In the case of *H. perforatum*, Scotti et al. also observed different chemical compositions of flower and leaf extracts in HPTLC [3]. According to the United States and European Pharmacopoeia, in the TLC of *H. perforatum,* the lower part should contain rutin, chlorogenic acid, and hyperoside, the middle part should contain three yellow bands of flavonoids, and the upper part should contain hypericin, pseudohypericin, and quercetin [34]. TLC finger print analysis indicated that the quality of *H. cordifolium* is somewhat similar to that of *H. perforatum* with some differences in the quantity of secondary metabolites. So, *H. cordifolium* can also be used as a herbal medicine like *H. perforatum.*

**Figure 1:**
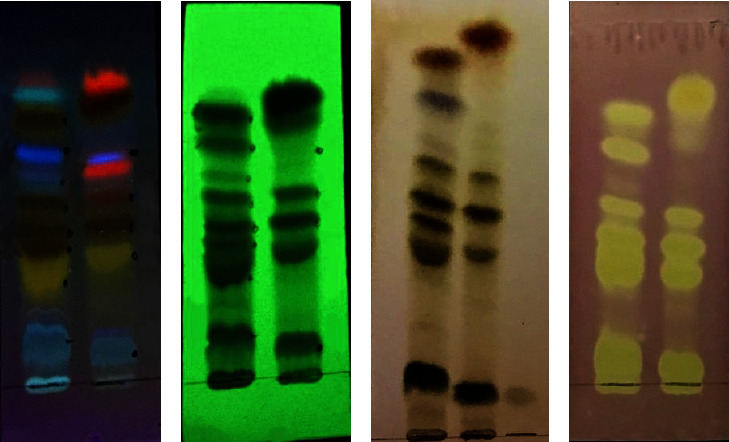
TLC of *H. cordifolium* flower (L) and leaf extract (R): (a–d) chromatograms visualized under UV lamp at 245, 366 nm, derivatized with FeCl_3_ and DPPH.

**Figure 2:**
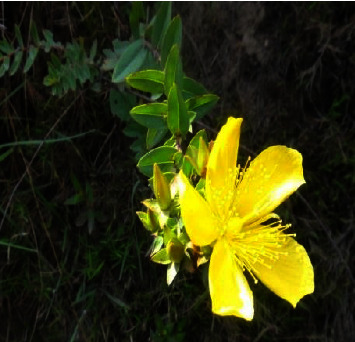
*H. cordifolium*.

The TLC plate after derivatizing with DPPH solution was analyzed in daylight. Yellowish bands on a purple background were marked. In flower extracts, bands of chlorogenic acid, mangiferin, hyperoside, quercetin, and other unidentified bands appeared on the TLC plate turned into yellow, whereas in the leaf extract, only a band of chlorogenic acid, three bands of flavonoids, and the uppermost band turned into yellow. The results of the TLC-DPPH assay revealed that the flower extract has more antioxidant phytocomponents than the leaf extract. The antioxidant activities of chlorogenic acid, mangiferin, hyperoside, and quercetin have been well reported [35–38]. The antioxidant activity of *H. cordifolium* could be due to the presence of these metabolites. The chromatograms visualized under 245, 366 nm, derivatized with FeCl_3_ and DPPH, are presented in Figures 1(a)–1(d), respectively. A photo of the plant is presented in Figure 2.

### 3.4. Total Phenolic and Flavonoid Contents

Polyphenols, mainly phenolic acids, flavonoids, and tannins are common in plant-derived foods and beverages. They showed the organoleptic properties of such foods and beverages [39]. As the antioxidant and other biological properties are directly related to the polyphenol content, it is necessary to estimate the total phenolics and flavonoids in plant samples. There are different methods for the determination of total phenolics. But the Folin–Ciocalteu method (F-C) is the most common and reliable method. In this method, in the alkaline medium, phenolic compounds transfer electrons to phosphomolybdic/phosphotungstic acid complexes to form blue complexes, possibly (PMoW_11_O_40_^4−^) that are determined colorimetrically at 760 nm [40, 41]. Gallic acid is widely used as the standard because it is very common in plants. This method gives a general measurement of phenolic content [42]. TPC in different extracts was calculated from the regression equation of the calibration curve (*Y* = 0.011*x* + 0.021; *R*^2^ = 0.994). In the case of leaf extracts, the TPC values are in the order, 50% aq methanol (261.25 mg GAE/g) > methanol (122.71 mg GAE/g) > ethylacetate (102.37 mg GAE/g). In the case of stem extracts, the TPC values are in the order, methanol (26.48 mg GAE/g) > 50% aq. methanol (24.06 mg GAE/g) > ethyl acetate (7.32 mg GAE/g). This indicated that the leaf extracts contained relatively higher amounts of phenolic compounds than stem extracts. In our previous work, we have found that the phenolic content of flower extracts was in the order, 50% aq. methanol (228.19 ± 0.639) > ethyl acetate (227.17 ± 0.736) > methanol (199.28 ± 0.576) [19]. This indicated that flower and leaf extracts are richer sources of phenolic compounds than stem extracts. Shresta et al. reported that the total phenolic content of the methanol extract of the leaf was 36.28 mg GAE/g dry extract which is much lower than our finding [43].

The total flavonoid content in plant extracts was determined colorimetrically using aluminum chloride. It is used as a complexing agent and form chelates of Al (III)-flavonoids due to their many oxo and hydroxyl groups. This assay was first developed by Christ and Muller for the determination of flavonol derivatives in drugs [44]. Later, it was modified by the addition of NaNO_2_ before the addition of AlCl_3_. It serves as a nitrating agent that is selective for aromatic vicinal diols to produce a flavonoid-nitroxyl derivative which shows absorption at 510 nm [24].

The total flavonoid content in different extracts was calculated from the regression equation of the calibration curve (*Y* = 0.002*x* + 0.002; *R*^2^ = 0.999). In the case of leaf extracts, the TFC values are in the order of methanol (232.60 mg CE/g) > 50% aq. methanol (62.63 mg CE/g) > ethyl acetate (51.47 mg CE/g). In the case of stem extracts, the TFC values are in the order of methanol (155.10 mg CE/g) > ethyl acetate (34.10 mg CE/g) > 50% aq. methanol (20.62 mg CE/g). In our previous work, we have found that the flavonoid content of flower extracts was in the order ethyl acetate (306.41 ± 0.95) > methanol (231.5 ± 0.93) > 50% aq. methanol (227.4 ± 0.99) [19]. This indicates that flower and leaf extracts are richer sources of flavonoid compounds than stem extracts. Shresta et al. reported that the total flavonoid content of the methanol extract of the leaf was 5.89 mg CE/g dry extract which is very much lower than our finding [43]. The low content of phenolics and flavonoids could be due to the chemical diversity arising from different collection sites and time. The results are shown in Table 4.

### 3.5. DPPH Free Radical Scavenging Activity

Plant phenolics have a preventive effect on chronic diseases caused by oxidative stress. It is well known that antioxidants such as vitamin C, vitamin E, carotenoids, organo sulfur compounds, and mostly polyphenols are very common in fruits, vegetables, nuts, and medicinal herbs. These antioxidants break radical chain reactions and prevent oxidative stress-related damage [45, 46]. Therefore, it is necessary to test the radical scavenging capacity of medicinal herbs. For this purpose, a stable DPPH free radical was used. When a solution of DPPH is mixed with a substance that can donate a hydrogen atom, DPPH is reduced with the loss of its violet colour to pale yellow.

In the DPPH free radical scavenging assay, the IC_50_ values of the leaf extracts were in the order, methanol (60.85 ± 2.67 *µ*g/ml) > 50% aq. methanol (63.09 ± 2.98 *µ*g/ml) > ethyl acetate (96.43 ± 3.85 *µ*g/ml). In the case of stem extracts, the order of IC_50_ value is methanol (89.39 ± 3.23 *µ*g/ml) > 50% aq. methanol and ethyl acetate extracts (>100 *µ*g/ml). In our previous work, we have found that the IC_50_ values of flower extracts were in the order, ethyl acetate (18.79 ± 0.98 *µ*g/ml) > methanol (18.98 ± 0.81 *µ*g/ml) > 50% aq. methanol (27.00 ± 1.03 *µ*g/ml) [19]. This indicated that the radical scavenging activity is in the order, flower > leaf > stem. This could be due to the presence of more phenolics and flavonoids in flower and leaf extracts than in stem extracts. This was also supported by the results of the TLC-DPPH^.^ analysis of flower and leaf extracts. In the DPPH free radical scavenging assay, the IC_50_ values of ethyl acetate (2.63 ± 0.01 *µ*g/ml), methanol (3.63 ± 0.01 *µ*g/ml), and methanol-water (4.71 ± 0.04 *µ*g/ml) extracts from aerial parts of *H. perforatum* have been reported by Öztürk et al. They found a strong correlation between higher phenolic/flavonoid content and antioxidant activities [42]. Similarly, Raut et al. reported the IC_50_ value (20.68 *µ*g/ml) of the methanol extract from the aerial parts of *H. cordifolium* in the DPPH free radical scavenging assay [47] which is close to our results for flower extracts as the aerial parts of *H. cordifolium* mostly contain flowers. The results are presented in Table 4.

### 3.6. Antimicrobial Activity

Bacterial infections and antibiotic drug resistance are the major challenges these days. Phytochemicals alone or in combination with antibiotics have exerted potential antibacterial activities against drug-sensitive and drug-resistant pathogens via different mechanisms of action [48–50]. Testing of plant extracts for antibacterial activity may help find new compounds that may cause synergistic effects with the existing antibiotics.

Flavonoids are known for their antibacterial activities [51, 52]. As the methanol extracts of the leaf and stem contained higher amounts of flavonoids than other extracts (Table 4), they were selected for antibacterial assay. The extracts were tested against one Gram-positive bacterium, *S. aureus,* and three Gram-negative bacteria, *E. coli*, *S. typhi*, and *S. sonnei*. It is well known that *S. aureus* is mainly responsible for skin infection, *S. typhi* for typhoid fever, while *E. coli* and *S. sonnei* for diarrhea and dysentery. The tested extracts showed only weak activities with the inhibition zones that ranged from 7 to 13 mm against all tested bacteria except for *S. typhi*. The antibacterial activities of the methanol extract of the leaf have been reported by Shrestha et al., against *S. aureus* and *E. coli*, as well as against some strains of multidrug resistance bacteria such as *K. pneumonia*, methicillin-resistant *S*. *aureus*, *P*. *aeruginosa*, and *A*. *baumannii* [43]. Similarly, Raut et al. reported the antibacterial activities of methanol extracts of aerial parts against *S. aureus* and *E. coli* [47]. The antibacterial activities depend on the season of collection, the plant part used, and the extraction methods [53]. Shrestha et al., and Raut et al., extracted the plant materials directly with methanol, and the overall antibacterial activity could be due to the acylphloroglucinols, naphthodianthrones, xanthones, phenolic acids, and flavonoids present in the extracts. Acylphloroglucinol derivatives have potential antibacterial and cytotoxic activities [54]. In our study, we have adopted the polarity-based extraction method where the antibacterial activity of the methanol extract could be due to polar phenolics and flavonoids compounds. These results of antibacterial activities support, to some extent, the traditional use of this plant to treat diarrhea, dysentery, and fever due to bacterial infections [16]. The results are presented in Table 5.

## 4. Conclusions

The present study highlights the phytochemical analysis and the antioxidant and antibacterial potential of leaf and stem extracts of *H. cordifolium*, native to Nepal. Qualitative TLC analysis of the methanol extract of the flower and leaf showed that they differ from each other in chemical composition. The flower extract showed the presence of more phytochemicals than the leaf extract. However, some common marker compounds such as chlorogenic acid and mangiferin were present in both extracts. Hyperoside and quercetin were present only in the flower extract. TLC finger print analysis revealed that the quality of *H. cordifolium* was somewhat similar to *H. perforatum*. In the TLC-DPPH^.^ assay, almost all phytochemicals of the flower extract showed radical scavenging activity, while only few phytochemicals in leaf extracts showed radical scavenging activity. Quantitative estimation of phenolics and flavonoids indicated that the leaf extracts were the good source of these phytochemicals than the stem extracts. In the antioxidant assay, the leaf extract showed lower IC_50_ values than the stem extracts. However, in statistical analysis using one-way ANOVA followed by the Tukey test, no significant difference between the IC_50_ values of the ethyl acetate extract of the leaf and the methanol extract of the stem was observed. A similar case was observed between the methanol and 50% aq. methanol extracts of the leaf. But there were significant differences in IC_50_ values between the extracts and standard ascorbic acid. In the antibacterial assay, the leaf extracts exhibited greater inhibition zones than the stem extracts. However, in statistical analysis, no significant difference in the inhibition zone was observed between the leaf and the stem extracts against *S. aureus* and *S. sonnei*. In contrast, significant differences were observed in the inhibition zones produced by extracts and the standard antibiotic, neomycin, against *S. aureus* and *S. sonnei*, while no difference was observed against *E. coli*. In conclusion, leaf and stem extracts of *H. cordifolium* were weak antioxidants and antibacterials in comparison to the respective standards. However, in general, the finding of our study indicated that in terms of the content of phytochemicals, antioxidant, and antibacterial potencies, the different anatomical parts of *H. cordifolium* are in the order flower > leaf > stem. Therefore, the underutilized natural resources of Nepal can be used for the formulation of dietary supplements, nutraceuticals, or herbal medications. But a toxicological study is also crucial before formulation of herbal drugs.

## Figures and Tables

**Table 1:** Percentage yield of leaf and stem extracts.

Extracts	Hexane	CH_2_Cl_2_	EtOAc	MeOH	50% aq. MeOH
Leaf	3.36	11.08	6.12	10.24	10.00
Stem	0.98	0.52	0.70	3.92	8.38

**Table 2:** Phytochemical screening of leaf and stem extracts.

Class of phytochemicals	Parts	Hexane	CH_2_Cl_2_	EtOAc	MeOH	50% aq. MeOH
Alkaloids	L	−	+	+	+	−
	S	−	−	−	−	−

Terpenoids	L	+	−	−	+	+
	S	+	+	+	+	+

Flavonoids	L	−	−	+	+	+
	S	−	−	+	+	+

Phenolics	L	−	+	+	+	+
	S	−	+	+	+	+

Glycosides	L	−	−	−	+	+
	S	−	−	−	+	+

Reducing sugars	L	−	−	−	+	−
	S	−	−	−	−	−

Saponins	L	−	−	−	+	+
	S	−	−	−	−	−

Tannins	L	−	+	+	+	+
	S	−	+	+	+	+

Quinones	L	+	+	+	−	−
	S	+	−	−	−	−

**Table 3:** TLC analysis of flower and leaf extracts in ethylacetate-methanol-water, 100 : 13.5 : 10 (v/v/v).

Band	Flower extract			Observed UV *λ*^max^ values in nm	Remarks	Leaf extract		
	Under 366 nm		Derivatized with FeCl_3_			Under 366 nm		Derivatized with FeCl_3_
	Colour	*R*_*f*_ values				Color	*R*_*f*_ values	
1	Blue	0.12	Green	329, 295 s, 245	Chlorogenic acid	Blue	0.12	Green
2	Light blue	0.20	Light green	—	Phenolic acid	Light blue	0.20	Green
3	Yellow	0.40	Green	368, 318, 260, 245	Mangiferin	Yellow	0.40	—
4	Brown	0.46	Green	361, 258, 209	Hyperoside	Not seen	—	Green
5	Brown	0.52	Green	—	Flavonoid	Brown	0.52	Green
6	—	—	—	—	Unidentified	Light pink	0.58	Not seen
7	Brown	0.60	Green	—	Flavonoid	Brown	0.60	Green
8	Blue	0.61	Light blue	—	Phenolic acid	Not seen	—	Not seen
9	Light pink	0.64	Not seen	—	Hypericin	Pink	0.64	Not seen
10	Blue	0.67	Not seen	—	Phenolic acid	Light pink	0.67	Not seen
11	Yellow	0.75	Blue	375, 255	Quercetin	Not seen	—	—
12	Brown	0.83	Brown		Unidentified	Not seen	—	—

**Table 4:** Total phenolic and flavonoid contents and antioxidant activity.

Extracts	Parts	EtOAc	MeOH	50% aq. MeOH	Ascorbic acid
Total phenolic content (mg GAE/g dry extract) (mean ± S.D) (*n* = 3)	L	102.37 ± 0.82	122.71 ± 2.40	261.25 ± 1.66	—
	S	7.32 ± 0.77	26.48 ± 2.02	24.06 ± 1.30	—

Total flavonoids content (mg CE/g extract) (Mean ± S.D) (*n* = 3)	L	51.47 ± 1.82	232.60 ± 10.52	62.63 ± 3.81	—
	S	34.10 ± 3.32	155.12 ± 4.30	20.60 ± 2.22	—

IC_50_ *µ*g/ml against DPPH free radical assay (mean ± S.D) (*n* = 2)	L	96.43 ± 3.85^*∗*°^	60.85 ± 2.67^*∗*•^	63.09 ± 2.98^*∗*•^	20.75 ± 0.58
	S	>100	89.39 ± 3.23^*∗*°^	>100	

Mean values of three experiments ± SD (^*∗*^*p* < 0.05 against positive control, ascorbic acid, ^•^*p* > 0.05 against each other, °*p* > 0.05 against each other).

**Table 5:** Antibacterial activity of the methanol extract.

Bacteria	Inhibition zone in mm		
	Plant parts		Standard antibiotic
	Leaf	Stem	Neomycin
*S. aureus*	13^*∗*°^	11^*∗*°^	23
*S. sonnei*	13^*∗*°^	7^*∗*°^	30
*E. coli*	12^•^	7^•^	16
*S. typhi*	—	—	24

(Inhibition zone measured including 6 mm well), mean values of three experiments. (^*∗*^*p* < 0.05 against the positive control, neomycin). (°*p* > 0.05 against each other). (^•^*p* > 0.05 against the positive control, neomycin).

## Data Availability

The data used to support the findings of the study are available from the corresponding author.
